# Factors associated with No-Shows and rescheduling MRI appointments

**DOI:** 10.1186/s12913-016-1927-z

**Published:** 2016-12-01

**Authors:** Majeed O. AlRowaili, Anwar E. Ahmed, Hasan A. Areabi

**Affiliations:** 1 King Abdullah International Medical Research Center (KAIMRC), College of Public Health and Health Informatics, King Saud bin Abdulaziz University for Health Sciences, Riyadh, Saudi Arabia; 2 Division of Magnetic Resonance Imaging, King Abdul-Aziz Medical City, Riyadh, Saudi Arabia; 3 Epidemiology and Biostatistics, College of Public Health and Health Informatics, King Saud bin Abdulaziz University for Health Sciences, MC 2350, P.O. Box 22490, Riyadh, 11426 Saudi Arabia

**Keywords:** MRI waiting times, No-Shows, Radiology services, Saudi Arabia

## Abstract

**Background:**

One of the major challenges facing global radiology services comes from delays connected to long waiting lists for magnetic resonance imaging (MRI) procedures. Such delays in diagnostic procedures could lead to poorer patient care outcomes. This study intended to estimate the rate of “No-Shows” or “Reschedule” MRI appointments. We also investigated the factors correlating No-Shows and Reschedule MRI appointments.

**Methods:**

A cross-sectional study was conducted in Saudi Arabia using data obtained via MRI schedule reviews and self-administrated questionnaires. Clinical and demographic data were also collected from the study participants. Stepwise binary logistic regression was used to analyze the data.

**Results:**

A total of 904 outpatients were asked to participate in the study, and we enrolled 121 outpatients who agreed to complete the study questionnaire. Of the 904 outpatients, the rate of No-Shows or Reschedule was 34.8% (95% Confidence Interval: 31.7–38.1%). Of the 121 outpatients studied, the rate of No-Shows or Reschedule was 49.6% (95% CI: 40.4–58.8%). Those of the female gender (OR = 6.238; 95% CI: 2.674–14.551, *p*-value = 0.001) and lack of education (OR = 2.799; 95% CI: 1.121–6.986, *p*-value = 0.027) were highly associated with No-Shows for the MRI appointments. There was no clarification of the MRI instructions (OR = 31.396; 95% CI: 3.427–287.644; *p*-value = 0.002), and family member drivers (OR = 15.530; 95% CI: 2.637–91.446, *p*-value = 0.002) were highly associated with rescheduling the MRI appointments.

**Conclusions:**

We noted higher rates of No-Shows and Rescheduling of MRI appointments in females, those with a lack of formal education, those who had not received the procedure instructions, and those who lacked transportation. We recommend setting targets and developing strategies and policies to improve more timely access to MRI, and thus reduce the waiting time.

**Electronic supplementary material:**

The online version of this article (doi:10.1186/s12913-016-1927-z) contains supplementary material, which is available to authorized users.

## Background

The use of diagnostic imaging devices has become universal in current medical diagnosis and healthcare services [1]. Long magnetic resonance imaging (MRI) procedure waiting lists constitute one of the major challenges facing radiology services worldwide. It has been well said that delays in diagnostic procedures leads to poorer patient care outcome and financial losses [2]. Further, MRI demand has increased significantly over the last few years [1, 3, 4], mainly driven by 1) MRI advancements to detect abnormalities, and 2) the non-invasive nature of MRI [1, 4]. MRI waiting lists are growing [4, 5]. The majority of those on the waiting lists are patients waiting for diagnosis, which calls for the attention of decision-makers to avoid delaying the start of treatment management [6, 7]. MRI procedures are performed for outpatients and inpatients, and urgency may be required to facilitate the MRI procedure in life-threatening clinical situations, e.g., patients with acute brain stroke [3, 5].

Many studies have identified long healthcare waiting times as one of the major challenges facing the healthcare system [2, 8–19]. It has been emphasized that these long waiting lists represent the most serious problem facing healthcare today. This was determined following a public opinion survey, which showed that 75% of the respondents identify reducing waiting lists as a high priority [10]. Some policymakers recommend solving the problem of waiting time by increasing resources [5, 10], while other studies show that upgrading the process would lead to improvement [2, 4]. Moreover, some researchers emphasize a multi-priority system to avoid the impact of a long waiting time for critical MRI patients. A number of models were concerned with the management of long waiting-time situations rather than solving the long waiting-list issues for all patients [20–24]. Several studies noted that management of inappropriate resource allocation may be one of the important keys to improving the diagnostic imaging waitlist system for MRI and thus improving quality of services [25–27].

Healthcare services are free to all Saudi Arabians. The Ministry of Health is responsible for overseeing the healthcare and hospital services in the public and private sectors. The Ministry of Health hospitals provide healthcare services to the public, and exist in large cities and villages across the Kingdom of Saudi Arabia.

Attendance at the MRI Department was identified as a key performance indicator for radiology [2, 5]. In healthcare, patient nonattendance as scheduled was closely linked to long waiting time, and it was concluded that long waiting time leads to high rates of no-show incidents, which, in turn, leads to increased waiting time [17, 18]. On the other hand, since being given an MRI scan requires pre-patient preparation, it is important to reschedule patients and cancel events. In addition, No-Shows lead to waste of slots and double occupancy for MRI scheduling slots. Rescheduling incidents were attributed to patient non-compliance and administrative reasons [28, 29].

It was concluded that No-Shows and Rescheduling patients lead to delay in treatment management and poorer patient-care outcome [29]. Most of the published studies in this regard focused on different strategies to overcome waiting for care [2, 6, 10, 11, 13] or evaluated the impact of long waiting periods for patients [7, 14]. In this study, we aimed to estimate the rate of No-Shows and Rescheduling for outpatient MRI appointments, the attributing factors, and the consequences because of the long waiting time.

## Methods

This is a cross-sectional study using an MRI facility as the main source of data about MRI-scheduled outpatients. The study was conducted in the Division of Magnetic Resonance Imaging at the King Abdul-Aziz Medical City-Riyadh (KAMC-R) from 5 August to 5 September 2014. The data from this study was collected by a student of the College of Public Health and Health Informatics, King Saud bin Abdulaziz University for Health Sciences, as part of the student’s degree requirements. King Abdul-Aziz Medical City-Riyadh has the most advanced devices for medical examinations to help diagnose diseases in the early stages. MRI examination is free to all citizens. Currently, the number of patients continues to grow, along with the demand for all types of examinations. The MRI equipment in this healthcare organization is shared between inpatients, outpatients, and emergency room patients. KAMC-R is striving for the unification of MRI standards, policies, and procedures according to international standards in order to provide high-quality services and reduce unnecessary procedures. In so doing, the conversion process becomes easier and shorter. The current study included outpatients scheduled for MRI who met the following inclusion criteria: 1) Able to give consent (18 years and older), and 2) Scheduled the MRI using the official scheduling system. Exclusion criteria were 1) Inability to speak English or Arabic, and 2) Lack of accessible medical charts or updated contact information. The study was approved by the IRB office at King Abdullah International Medical Research Center, Riyadh, Saudi Arabia.

**Fig. 1:**
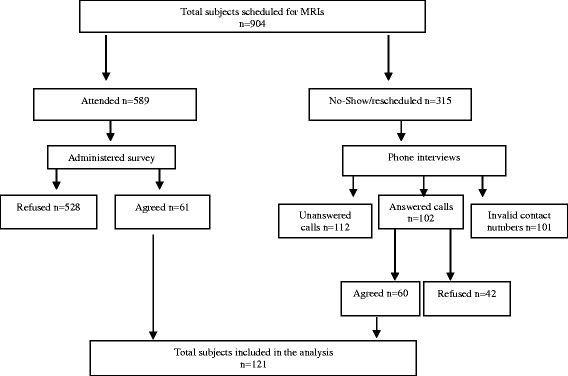
The study flow chart of sample selection

The intended goal was to collect demographic and other qualitative data using self-administrated questionnaires developed and tested by the researchers. Initially, the questionnaires were distributed to 20 outpatients visiting the MRI department in order to evaluate the questionnaire’s clarity and validity. After reviewing the responses, the researchers modified the questionnaire accordingly. All subjects who were scheduled for MRIs between 5 August and 5 September 2014 were included in the study. A total of 904 subjects were scheduled for MRIs during the study period, and 589 subjects appeared for their MRI appointments, as indicated in the flow chart (Fig. 1). These subjects were given the study questionnaire to complete (528 refused to complete the questionnaire and 61 complied). There were 315 who did not show up or who had rescheduled their MRI appointments. These 315 subjects had phone interviews (there were 112 unanswered calls in three attempts, 102 answered calls, and 101 invalid contact numbers). Of 102 subjects who answered calls, 42 refused and 60 agreed to complete the interview. A final total of 121 subjects were included in the analysis.

The questionnaire consisted of two parts (Additional file 1). The first part reflected concern about the patients’ demographic data, and the second part contained specific questions related to the MRI scheduling procedure, instructions procedure, methods of communication, and transportation to the hospital. Participants who did not show were asked to give a reason for not appearing for their MRI as scheduled.

### Statistical analyses

Data were analyzed using SPSS® V. 23.0 (SPSS, Chicago, Illinois, USA). All scheduled MRI outpatients during the period from 5 August to 5 September were classified into three groups: 1) Attended and completed the procedure, 2) Attended but rescheduled, and 3) No-Shows. Descriptive statistics were used to summarize demographic/clinical characteristics (Tables 1). Chi-square tests were used for the associations between demographic/clinical characteristics and MRI No-Shows (Table 2). The chi-square tests were also used for the associations between demographic/clinical characteristics and MRI Rescheduling (Table 3). Means and standard deviations (*mean ± SD*) were used to summarize patients’ ages. Effects of age were assessed across the MRI No-Show (Table 2) and Reschedule groups (Table 3) using t-tests. Stepwise logistic models were employed to identify the risk factors associated with No-Shows (Table 4) and Rescheduling (Table 5). The strength of the relationship was assessed using odds ratios (OR) and 95% confidence intervals (CI).

**Table 1:** Sample characteristics, *N* = 121

Characteristics	Levels	*n*	%
Age	Mean (± SD) Years		39 ± 18
Type of incidents	No-Show	45	37.2
	Show/MRI performed	61	50.4
	Show/MRI Rescheduled	15	12.4
Gender	Male	79	65.3
	Female	42	34.7
Nationality	Saudi	116	95.9
	Non-Saudi	5	4.1
Living area	In Riyadh	93	76.9
	Outside Riyadh	28	23.1
Marital status	Married	97	80.2
	Unmarried	24	19.8
Schedule clarified	Yes	103	85.1
	No	18	14.9
Procedure’s instruction clarified	Yes	69	57.0
	No	52	43.0
Communication	My phone	65	53.7
	Others	56	46.3
Transportation	Driving	50	41.3
	Self-drive/Taxi	15	12.4
	Family member driver	56	46.3
Monthly income	SR10,000 or less	83	68.6
	More than SR10,000	38	31.4
Education	High school or less	78	64.5
	Bachelor’s or above	43	35.5

**Table 2:** Association between No-Show and demographic and clinical characteristics, *N* = 121

		No-Show 45 (37.2%)		Show 76 (62.8%)		*P*
Characteristic	Levels	*N*	%	*N*	%	*P*
Age	Mean (± SD) Years	41 (±20.4)		37.7 (±16.0)		0.324
Gender	Male	18	22.8	61	77.2	0.001*
	Female	27	64.3	15	35.7	
Nationality	Saudi	45	38.8	71	61.2	0.079
	Non-Saudi	0	0.0	5	100.0	
Living area	In Riyadh	40	43.0	53	57.0	0.016^*^
	Outside Riyadh	5	17.9	23	82.1	
Marital status	Married	34	35.1	63	64.9	0.328
	Unmarried	11	45.8	13	54.2	
Schedule clarified	Yes	41	39.8	62	60.2	0.154
	No	4	22.2	14	77.8	
Procedure’s instruction clarified	Yes	23	33.3	46	66.7	0.312
	No	22	42.3	30	57.7	
Communication	My phone	18	27.7	47	72.3	0.020^*^
	Others	27	48.2	29	51.8	
Transportation	Self-drive	14	28.0	36	72.0	0.022^*^
	Taxi	3	20.0	12	80.0	
	Family member	28	50.0	28	50.0	
Monthly income	10,000 or less	33	39.8	50	60.2	0.388
	More than 10,000	12	31.6	26	68.4	
Education	High school or less	35	44.9	43	55.1	0.019^*^
	Bachelor’s or above	10	23.3	33	76.7	

^*^The chi-square statistic is significant at α = .05 level. Show is defined as attended and MRI performed or rescheduled

**Table 3:** Association between MRI rescheduled and demographic and clinical characteristics in patients who showed up for their MRI appointments (*n* = 76)

		Show/MRI performed 61 (80.3%)		Show/MRI rescheduled 15 (19.7%)		
Characteristic	Levels	*N*	%	*N*	%	*P*
Age	Mean (± SD) Years	36 (±12)		46 (±26)		0.031^#^
Gender	Male	53	86.9	8	13.1	0.008^*^
	Female	8	53.3	7	46.7	
Nationality	Saudi	57	80.3	14	19.7	0.678
	Non-Saudi	4	80.0	1	20.0	
Living area	In Riyadh	38	71.7	15	28.3	0.002^*^
	Outside Riyadh	23	100.0	0	0.0	
Marital status	Married	51	81.0	12	19.0	0.498
	Unmarried	10	76.9	3	23.1	
Schedule clarified	Yes	47	75.8	15	24.2	0.033^*^
	No	14	100.0	0	0.0	
Procedure’s instruction clarified	Yes	45	97.8	1	2.2	0.001^*^
	No	16	53.3	14	46.7	
Communication	My phone	44	93.6	3	6.4	0.001^*^
	Others	17	58.6	12	41.4	
Monthly income	10,000 or less	42	84.0	8	16.0	0.201
	More than 10,000	19	73.1	7	26.9	
Education	High school or less	32	74.4	11	25.6	0.120
	Bachelor’s or above	29	87.9	4	12.1	
Transportation	Self-drive/Taxi	46	95.8	2	4.2	0.001^*^
	Family Member	15	53.6	13	46.4	

^#^The *t*-test statistic is significant at α = .05 level

^*^The chi-square statistic is significant at α = .05 level

**Table 4:** Risk factors of No-Show using stepwise logistic regression (*N* = 121)

					95% CI for OR	
Characteristic	Reference	B	P	OR	Lower	Upper
Female	Male	1.831	0.001	6.2	2.674	14.551
High school education or less	Bachelor’s or above	1.029	0.027	2.8	1.121	6.986
Constant		−1.927	0.001	0.15

**Table 5:** Risk factors of MRI Rescheduled using stepwise logistic regression (*N* = 76)

					95% CI for OR	
Characteristic	Reference	B	P	OR	Lower	Upper
No procedure instruction	Procedure instruction	3.45	0.002	31.4	3.427	287.644
Family member driver	Self-drive/Taxi	2.74	0.002	15.5	2.637	91.446
Constant		−5.26	0.001	0.01		

## Results

The 904 outpatients scheduled for MRIs during the study period were included in the initial analysis. The mean age of these patients was 40.14 ± 20, and 55% were females. Of outpatients studied, 589 (65.1%) checked in and completed the MRI, 36 (4.0%) checked in but rescheduled, and 279 (30.9%) missed their MRI appointments. The rate of No-Shows or Rescheduling was 34.8% (95% CI: 31.7–38.1%). Of the 904 outpatients, 121 agreed to complete a survey as part of their MRI schedule.

The demographic and clinical characteristics of these 121 patients are shown in Table 1. Of these patients, 61 (50.4%) completed the MRI procedure, 45 (37.2%) were No-Shows, and 15 (12.4%) rescheduled their MRI appointments. The rate of No-Shows or Rescheduling was 49.6% (95% CI: 40.4–58.8%), 103 (85.1%) reported that the MRI schedule was clarified, 69 (57%) reported that they received the procedure instructions, 65 (53.7%) used their own mobile phones, and 56 (46.3%) used family members’ mobile phones.

The associations between No-Shows and demographic/clinical characteristics were analyzed using chi-square tests and independent t-tests as shown in Table 2. The high rate of MRI No-Shows was significantly associated with the female gender (64.3% in women vs. 22.8% in men, *p* = 0.0001). Interestingly, we noted a high rate of No-Shows in patients residing in Riyadh compared to patients residing outside of Riyadh (43% vs. 17.9%, *p* = 0.016). The rate of No-Shows was lower in patients who used their own mobile phones compared to those who used family members’ mobile phones (27.7% vs. 48.2%, *p* = 0.020). No-Shows were significantly less frequent in patients who used a driver/taxi compared to those who drove themselves or used family members as drivers (20% vs. 28% and 50%, *p* = 0.022). A higher rate of No-Shows was observed in patients with high school education or less compared to patients with a bachelor’s degree or above (44.9% vs. 23.3%, *p* = 0.019).

The associations between the demographic and clinical characteristics and rescheduling MRI appointments are shown in Table 3. The patients who rescheduled their MRIs were older compared to those who showed up and completed the MRIs (46 ± 26 vs. 36 ± 12 years, *p* = 0.031). The high rate of rescheduling was associated with the female gender (46.7% in women vs. 13.1% in men, *p* = 0.008). The rate of rescheduling was higher in patients residing in Riyadh compared to patients residing outside of Riyadh (28.3% vs. 0.0%, *p* = 0.002). A lower rate of rescheduling was noted in patients who received instructions and clarification about the procedure than in those who did not (2.2% vs. 46.7%, *p* = 0.001). The rate of rescheduling was lower in patients who used their own mobile phones compared to those who used family members’ mobile phones (6.4% vs. 41.4%, *p* = 0.001) and was significantly lower in patients who drove themselves or used taxis compared to those who used family members as drivers (4.2% vs. 46.4 and 50%, *p* = 0.001).

Risk factors of No-Shows were identified by a stepwise logistic regression model (Table 4). The high rate of No-Shows was significantly associated with the female gender and those with high school education or less. We noted that the odds of No-Shows were six times higher in women than those in men (OR: 6.2; 95% CI: 2.674–14.551). The odds of No-Shows in patients with high school education or less were approximately three times higher compared to patients with a bachelor’s degree or above (OR: 2.8; 95% CI: 1.121–6.986). Similarly, we found two factors associated with a high rate of rescheduling (Table 5). The patients who did not receive proper clarification about procedures were 31 times more likely to reschedule than those who received proper clarification about the procedure’s instructions (OR: 31.4; 95% CI: 3.427–287.644). The odds of rescheduling were 15 times higher in patients with a family driver compared to self-drive/taxi (OR: 15.5; 95% CI: 2.637–91.446).

## Discussion

Waiting time as emphasized by other public surveys is one of the major challenges facing the healthcare system [7, 8]. This study evaluated the impact of patients’ No-Shows and rescheduling on the MRI waiting list, and whether or not demographic and socio-economic factors were associated with No-Shows and Rescheduling. A total of 904 outpatients were scheduled for MRIs during the study period. We chose this time to avoid the effect of seasonal holidays and Ramadan, when No-Shows rates are even higher. Our study shows that nearly 35% of scheduled outpatients either missed their appointments or rescheduled for various reasons.

We found that gender influences patient appearance for the MRI appointment. Women were six times more likely than men to not show on the day of the MRI. The effect of cultural barriers – since women in the studied sample are not allowed to drive – provides an explanation for this phenomenon. Contrary to previous studies [30, 31], patients with high school education or less were about three times more likely to be No-Shows than those with a bachelor’s degree or above. Furthermore, patients who were unable to provide personal phone numbers to receive SMS (text) reminders from the hospital, and who were unable to secure transportation to the hospital were more likely to not check in as scheduled. This could be due to cultural, economic, or social reasons; however we were unable to collect such information.

Rescheduled outpatients comprised nearly 5% of the total scheduled outpatients during the study period. Most were due to administrative reasons, which is similar to a study conducted on elective surgery patients in 1990 [29]. Only 15 out of the 36 Rescheduled patients agreed to participate in our study. We found the majority of those patients did not receive proper instructions while originally requesting and scheduling the MRI. Results showed that procedure instructions were not clarified for 93.3% of the patients who were rescheduled vs. 26% of the patients who managed to complete the MRI. Our analysis suggests that patients who had not received clarification of the MRI instructions were 31 times more likely than other patients to be rescheduled. This result emphasizes the conclusion of a previous study on patients scheduled for endoscopy [28]. Furthermore, our study shows that patients who need transportation to the hospital are 15 times more likely to be rescheduled. This could be due to the fact that most of those patients are uneducated older adults. Our study shows that the main reason for male patients not attending was being at work (66.7%), and for women, it was lack of transportation (44.4%). Saudi Arabia applies social and strict standards and is the only country in the world that prohibits women from driving.

It is worth mentioning that during the study, we had difficulty contacting patients by phone. Out of the 315 patients who either did not show or rescheduled, we managed to contact only 102: nearly 32% were wrong numbers, and the rest did not answer after three attempts. This raises a concern about the validity of the available contact numbers. Some of our patients might not receive the reminders from the hospital SMS system in the first place, and so forgot their appointments. Stubbs et al. emphasized that text messages are the most effective method to remind patients about their appointments and minimize the no-shows [24]. We could not validate the contact numbers for all patients due to a shortage of resources and time.

These findings warrant consideration to the study limitations. Some are rising from the nature of the study itself. Also, a drawback is the inability to validate the contact number for all participants who might contribute to high rates of No-Shows. In addition, we were unable to include the chief patient complaint to the list of possible risk factors, as we were short of resources. However, the strength of this study is its attention to patient input within one week of the scheduled date. To the best of our knowledge and after literature review, we believe that this study is the first one discussing factors associated with no-shows and rescheduling, and their impact on MRI waiting lists in Saudi Arabia.

## Conclusions

Considerably higher rates of No-Shows and rescheduling MRI appointments were observed in our hospital, particularly among the female gender, those with a lack of formal education, those who had not received the procedure instructions, and those who lacked transportation. These findings suggest that implementing effective hospital strategies and policies to improve access to MRI and reduce the waiting time are needed. This may include training the schedulers, setting targets, confirming attendance one or two days prior to the MRI appointment, frequent updates of contact information, and supported mechanisms targeting the disadvantaged socio-economic group.

## Additional file

Additional file 1: The Study Questionnaire. (DOC 36 kb)

## Abbreviations

CI: Confidence interval
KAIMRC: King Abdullah International Medical Research Center
KAMC-R: King Abdul-Aziz Medical City-Riyadh
MRI: Magnetic resonance imaging
OR: Odds ratio
