# Supplementary material for: Factors associated with No-Shows and rescheduling MRI appointments
Source: BMC Health Serv Res. 2016 Dec 1;16:679. doi: 10.1186/s12913-016-1927-z (PMC5133747; doi:10.1186/s12913-016-1927-z)
Supplement: Additional file 1: — The Study Questionnaire. (DOC 36 kb) [file 12913_2016_1927_MOESM1_ESM.doc]

**Questionnaire**

All responses will be kept confidential and anonymous.

Thank you for your time.

**A) General Questions**

1. **Are you the patient?**
2. Yes
3. No, Please specify the relationship….
4. **Age ……….**Years
5. **Gender**
6. Male
7. Female
8. **Nationality**
9. Saudi
10. Non Saudi
11. **Marital Status**
12. Married
13. Unmarried
14. **Education**
15. High school or less
16. Bachelor or above
17. **Living Area**
18. In Riyadh
19. Outside Riyadh.
20. **Monthly income in Saudi Riyal.**
21. SR10,000 ($2666.7) or less
22. More than SR 10,000 ($2666.7)

**B) Questions related to the appointment in MRI**

1. **Have the procedures’ instructions and preparation been clarified to you?**
2. Yes
3. No
4. **Transportation to Hospital.**
5. Self-drive
6. Taxi
7. Family member
8. **How MRI appointment communicated with you?**
9. Personal mobile phone.
10. Others, Specify
11. **Type of incidents**
12. No-Show
13. Show/MRI performed
14. Show/MRI Rescheduled
15. **In case of No show, reasons attributed to no show.**
16. Forget the appointment.
17. Busy at work.
18. Illness.
19. No Transportation.
20. Long distant.
21. Others, Specify…….
